# Military-Related Exposures, Social Determinants of Health, and Dysbiosis: The United States-Veteran Microbiome Project (US-VMP)

**DOI:** 10.3389/fcimb.2018.00400

**Published:** 2018-11-19

**Authors:** Lisa A. Brenner, Andrew J. Hoisington, Kelly A. Stearns-Yoder, Christopher E. Stamper, Jared D. Heinze, Teodor T. Postolache, Daniel A. Hadidi, Claire A. Hoffmire, Maggie A. Stanislawski, Christopher A. Lowry

**Affiliations:** ^1^Rocky Mountain Mental Illness Research Education and Clinical Center, Rocky Mountain Regional VA Medical Center (MIRECC), Aurora, CO, United States; ^2^Department of Physical Medicine & Rehabilitation, University of Colorado Anschutz Medical Cam pus, Aurora, CO, United States; ^3^Departments of Psychiatry and Neurology, University of Colorado Anschutz Medical Cam pus, Aurora, CO, United States; ^4^Military and Veteran Microbiome: Consortium for Research and Education, Aurora, CO, United States; ^5^Department of Systems Engineering, Air Force Institute of Technology, Wright-Patterson AFB, OH, United States; ^6^Department of Integrative Physiology, University of Colorado Boulder, Boulder, CO, United States; ^7^Mood and Anxiety Program, University of Maryland School of Medicine, Baltimore, MD, United States; ^8^VISN 5 MIRECC, Department of Veterans Affairs, Baltimore, MD, United States; ^9^Department of Epidemiology, University of Colorado Anschutz Medical Cam pus, Aurora, CO, United States; ^10^Center for Neuroscience, University of Colorado Boulder, Boulder, CO, United States; ^11^Center for Neuroscience, University of Colorado Anschutz Medical Cam pus, Aurora, CO, United States

**Keywords:** microbiome, Veteran, dysbiosis, psychological and physical health, mental health

## Abstract

Significant effort has been put forth to increase understanding regarding the role of the human microbiome in health- and disease-related processes. In turn, the United States (US) Veteran Microbiome Project (US-VMP) was conceptualized as a means by which to serially collect microbiome and health-related data from those seeking care within the Veterans Health Administration (VHA). In this manuscript, exposures related to military experiences, as well as conditions and health-related factors among patients seen in VHA clinical settings are discussed in relation to common psychological and physical outcomes. Upon enrollment in the study, Veterans complete psychometrically sound (i.e., reliable and valid) measures regarding their past and current medical history. Participants also provide skin, oral, and gut microbiome samples, and permission to track their health status via the VHA electronic medical record. To date, data collection efforts have been cross-diagnostic. Within this manuscript, we describe current data collection practices and procedures, as well as highlight demographic, military, and psychiatric characteristics of the first 188 Veterans enrolled in the study. Based on these findings, we assert that this cohort is unique as compared to those enrolled in recent large-scale studies of the microbiome. To increase understanding regarding disease and health among diverse cohorts, efforts such as the US-VMP are vital. Ongoing barriers and facilitators to data collection are discussed, as well as future research directions, with an emphasis on the importance of shifting current thinking regarding the microbiome from a focus on normalcy and dysbiosis to health promotion and disease prevention.

## Introduction

The Human Microbiome Project (HMP) was launched with the goal of characterizing genetic material derived from microbes that inhabit the human body, and designed to increase understanding regarding the “microbial components” of the human “genetic and metabolic landscape, and how they contribute to our normal physiology and disease predisposition” (Turnbaugh et al., 2007). Initial efforts included sequencing microbiomes at multiple sites of 300 “healthy” human subjects (Turnbaugh et al., 2007). By design, HMP participants lacked evidence of disease (dpGaP, 2011).

More recently, a large-scale human microbiome study was completed by researchers associated with the American Gut Project (AGP) (McDonald et al., 2018). Instead of purposefully recruiting “healthy” adults, AGP investigators employed a novel citizen science-based methodology. Surveys created by the investigative team were used to obtain information regarding self-assessed health status, disease history, and lifestyle data. Initial analyses based on data from the AGP also have been focused on the “healthy adult, subset” (McDonald et al., 2018, p. 4). Individuals included in the analysis (*n* = 3,942) ranged in age from 20 to 69, and were highly educated and of relatively high socioeconomic status in relation to the general population.

Aided by the landmark HMP study, researchers have started investigating the human microbiome and its relation to clinical conditions including obesity (Ley et al., 2006), Alzheimer's disease (Hill et al., 2014), cancer (Jobin, 2018), and mental health (Stamper et al., 2016; Qiao et al., 2018). While one of the many deliverables from the HMP was to provide a reference dataset from a “healthy” cohort, efforts are needed to delineate features of microbial communities among those seeking medical services. Because of the diversity of the patient population and the availability of data from the electronic medical records (EMRs) the Veterans Health Administration (VHA) is an optimal setting to engage in such work.

According to the Department of Veterans Affairs, the US Veteran population is projected to include 20 million individuals in 2017 (National Center for Veterans Analysis and Statistics, 2016). A Veteran is “a person who served in the active military, naval, or air service, and who was discharged or released under conditions other than dishonorable,” (Code of Federal Regulations, 2014). At present, the largest cohorts of Veterans by conflict include those who served in the Gulf War Era (7,271,000), followed by those who served in Vietnam (6,651,000). These numbers highlight the bimodal age distribution of those seeking VHA care (National Center for Veterans Analysis and Statistics, 2016).

Overall, Veterans are predominantly male, and male Veterans are older than female Veterans, with median ages of 65 and 41, respectively (National Center for Veterans Analysis and Statistics, 2018). The numbers of minority and female Veterans continue to increase. By 2037, it is expected that minority Veterans will comprise 32.8% of the total Veteran population, as compared to the current 23.2% (National Center for Veterans Analysis and Statistics, 2016). In 2015, women represented ~9% of the total Veteran population, for a total of 2 million women Veterans; however, over the next 10 years, the population of women Veterans is expected to increase by 18,000 per year (National Center for Veterans Analysis and Statistics, 2017). In Fiscal Year 2015, 6 million (~30%) Veterans used their health care benefits (National Center for Veterans Analysis and Statistics, 2015). Use of such benefits varies by cohort and is contingent on many factors including age, gender, and household income.

In terms of access to historic medical data, the VHA was one of the first healthcare systems to adopt EMRs. The VHA's Corporate Data Warehouse (CDW) is a national repository, in which data from both the Veterans Information Systems and Technology Architecture (VISTA) and other VHA clinical and administrative systems are compiled. Complete medical data (e.g., appointment encounter codes, medications prescribed) are available from October 1999 forward. Records include care obtained within the VHA, as well as services received outside (private providers), but paid for by the VHA. Data from the CDW can also be linked with Medicare data. As such, data available within the CDW are frequently used to facilitate large-scale studies of both Veterans and the VHA health care system. The VHA also has a growing biobank of genetic information on Veterans through the Million Veteran Program (U.S. Department of Veterans Affairs, Office of Research & Development, 2018) (with more than 600,000 US Veterans enrolled as of September, 2017), which aims to leverage the longitudinal patient information available in the VHA in order to study the genetic influences on health and disease (Gaziano et al., 2016).

Those who served in the US Armed Forces, and particularly those who deployed to combat zones, have unique histories of physical and psychological exposures that have been shown to negatively impact current functioning. Data suggest that such exposures place individuals at increased risk for future negative physical (Ahmadi et al., 2011) and psychological outcomes (Brenner et al., 2011). Moreover, Veterans seeking VHA care have a higher prevalence of diagnosed health conditions than both non-Veterans and Veterans not seeking VHA care. According to work by Eibner et al. (2016), Veterans are 13.5 times more likely than non-Veterans to have posttraumatic stress disorder (PTSD). In fact, about a quarter of all who sought VHA care had a mental health diagnosis, with rates of some conditions being much higher among younger cohorts (Eibner et al., 2016). Moreover, those who seek care within the VHA are generally older and of a lower socioeconomic status than Veterans who do not use VHA care (Eibner et al., 2016). The average household incomes of VHA patients (2006–2012) are more than 20% lower ($35,981) than those who do use VHA care ($45,278). Similarly, those who use VHA care are less likely to be employed than those who do not use such care, 41.3% and 62.8%, respectively (Eibner et al., 2016).

Exposures and health conditions that will be measured among this cohort have been linked to the microbiome, such as disrupted sleep, medication use, obesity, and PTSD (Turnbaugh et al., 2009; Zhao, 2013; Falony et al., 2016; Hemmings et al., 2017; Reynolds et al., 2017). For example, the AGP team analyzed a subset (*n* = 125) of participants with mental illness (depression [97% of the clinical population], PTSD [10%], bipolar disorder [4%], and schizophrenia [2%]) against 1:1 matched healthy controls (*n* = 125) and found differences in gut microbiome community composition (McDonald et al., 2018). It should be noted that such diagnoses were based on responses to self-report questions. In addition, Hemmings et al. (2017) found that relative to 12 trauma-exposed controls, 18 participants with PTSD were identified as having different microbiome community composition.

## United states-veteran microbiome project (US-VMP)

Considering both the unique nature of the VHA Veteran cohort, as well as opportunities provided by a high quality single payer health care system (e.g., nation-wide EMR), members of this team began serially collecting microbiome and health-related data from those eligible to seek care within the VHA. Here we report on processes and procedures for obtaining the skin, oral, and gut microbiomes of an initial cohort of 188 US Veterans who have been enrolled in the US-VMP. In addition to the microbiome samples, extensive information was collected from participants via the administration of psychometrically sound measures regarding physical and mental health (described below). Veterans participating in this study also provided the investigative team with permission to access their VHA EMR. Within such records, a wealth of information on medical and medication history, as well as medical follow-up and outcomes over time is documented. We also describe several additional psychometrically sound measures that were recently implemented along with serial sampling procedures that will allow for longitudinal analyses. Included among the newly-deployed measures is a tool regarding the built environment that members of our team have developed and will be validating. For more information regarding the microbiome of the built environment and health, see Hoisington et al. (2015) and Stamper et al. (2016).

All US Veterans are eligible to enroll in this study. The month of first enrollment was May 2016. Prior to March 12, 2018, observational data were collected at a single time point for each participant. However, permissions have since been received to serially sample consented participants at 6-month intervals. Veterans who consented prior to 3/12/18 will also be notified regarding the opportunity to provide data and samples serially at 6, 12, 18, or 24 months following their initial consent date. Baseline visits have always been conducted in person. This will continue to be the case. In terms of the health-related measures described below, serial 6-month assessments can be conducted in-person, by phone, or via internet survey. Post-baseline microbiome samples can also be collected in person, or sent in by the participant, who will self-collect the samples at home. Demographic, military, and psychiatric history data will be provided below on the first 188 Veterans enrolled. All research and experimental practices associated with this study were approved and performed in accordance with the Colorado Institutional Review Board, as well as the local VA review committee.

Baseline skin, oral, and fecal samples were collected with double-tipped polyurethane swabs (BD BBL™ CultureSwab™ EZ II, Cat No. B220144, Fisher Scientific, Pittsburgh, PA, USA). Baseline skin and oral swabs were collected by the participant during an inpatient evaluation and were stored at −80 °C. Baseline skin samples were collected by swabbing the antecubital fossa (inner elbow), a body site characteristically involved in atopic dermatitis, an autoimmune disease, and consistent with a number of previous studies (Grice et al., 2008; Kong et al., 2012; Oh et al., 2012). Oral samples were collected by swabbing the buccal mucosa (inner cheek), a location selected to match HMP sites (Grice et al., 2009). Participants either provided a fecal sample during the same baseline visit as when the skin and oral samples were collected (*n* = 67) or received a pre-packaged sampling kit for home use with instructions for sample collection (*n* = 121). All samples collected at home were placed in residential freezers immediately and mailed back to the Rocky Mountain Mental Illness Research, Education and Clinical Center (MIRECC) in a pre-labeled package containing an ice pack. Current preservation includes the unused swab and the extracted DNA from all samples. Future work could include preservation of viable microbial communities and production of fecal microbiome transplant pills. Detailed protocols for how skin, oral, and fecal samples were acquired can be found in Appendix 1. Negative controls included swabs that were never opened (manufacturing control) or opened in the clinic but never used for sampling (clinical control). Upon receipt, all the samples were stored at −80°C. All samples were driven ~20 miles to the University of Colorado Boulder for molecular processing using a portable freezer, which allowed for the samples to be stored at −19°C during transportation. In the future, skin and oral microbiome samples may also be collected by the Veterans in their homes. They will be taught how to collect such samples during the baseline visit and will return samples as described above.

To obtain health-related information, efforts were made to select psychometrically sound tools frequently used to evaluate Veteran cohorts. During the in-person baseline clinical visit, participants completed the measures outlined in Table 1. Additional information regarding the measures is provided below. As of March 2018, survey measures are re-administered every 6 months. In addition to the collected measures, the VHA EMR contains longitudinal historical information regarding Veteran height and weight, as well as data from inpatient, outpatient, and fee-for-service medical encounters across the following domains: diagnoses, prescriptions and procedures, and laboratory orders and results. Information about substance use (e.g., opioid and recreational drug use) is also gathered from the EMR.

**Table 1:** Measures administered.

**Measure**	**Time to administer (minutes)**	**Condition(s)/Factor(s) of Interest**
**RISK ASSESSMENT**		
University of Washington Risk Assessment Protocol-Revised (UWRAP)	5	Risk assessment
**STRUCTURED CLINICAL INTERVIEWS**		
Ohio State University Traumatic Brain Injury (TBI) Identification Method (OSU TBI-ID)	25	Lifetime history of traumatic brain injury
Structured Clinical Interview for DSM-5-TR Axis I Disorders, Research Version, Patient Edition with Psychotic Screen (SCID-5-I/P W/PSY SCREEN)	30	Current and past psychiatric history
**SELF-REPORT MEASURES INCLUDED IN THE ORIGINAL PROTOCOL**		
Beck Depression Inventory (BDI-II)^*^	5	Depression-related symptoms
Harvard Food Frequency Questionnaire 2007 Booklet (Harvard FFQ)	20	Macro and micronutrient quantities
Insomnia Severity Index (ISI)^*^	5	Insomnia symptoms
Outcome Questionnaire-45 (OQ-45)^*^	10	Psychological distress
PTSD Checklist for DSM-5 (PCL-5)^*^	5	Posttraumatic symptoms
Rocky Mountain MIRECC Demographics Questionnaire	5	Personal and military characteristics
36-Item Short Form Health Survey (SF-36)^*^	10	Perceived health (general, physical/mental health)
**SELF-REPORT MEASURES ADDED TO THE PROTOCOL MARCH 2018**		
International Physical Activity Questionnaire (IPAQ) Short Form^*^	5	Physical activity
Morningness/Eveningness Questionnaire (MEQ)^*^	5	Individual chronotype
National Health Interview Survey (NHIS) – Chronic Conditions^*^	5	Chronic health conditions
Neurobehavioral Symptom Inventory (NSI)^*^	5	Postconcussive characteristics
Patient Health Questionnaire-9 (PHQ)-9^*^	5	Depression-related symptoms
Seasonality Pattern Assessment Questionnaire (SPAQ)^*^	5	Seasonality of mood and behavior; Seasonal Affective Disorder
**SELF-REPORT MEASURE REGARDING THE BUILT ENVIRONMENT ADDED TO THE PROTOCOL MARCH 2018**		
Housing, Occupancy, Materials, and Environment (HOME) Survey	5	Aspects of the built environment that influence individual mental well-being
**INFORMATION FOR ELECTRONIC MEDICAL RECORD/SELF-REPORT/MEASURED IN PERSON WHEN POSSIBLE**		
Height and Weight^*^	2	Body-mass index

* *Administered at baseline and during serial follow-up assessments, every 6 months post-baseline (serial follow-up assessment began in March, 2018)*.

### Measures

#### Risk assessment

University of Washington Risk Assessment Protocol-Revised (UWRAP). The UWRAP is used to assess and address any potential risk associated with participating in the study (Reynolds et al., 2006).

#### Structured clinical interviews

Ohio State University TBI Identification Method (OSU TBI-ID). The OSU TBI-ID is a structured interview for the detection of history of exposure to TBI (Corrigan and Bogner, 2007; Bogner and Corrigan, 2009).

Structured Clinical Interview for DSM-5-TR Axis I Disorders, Research Version, Patient Edition with Psychotic Screen (SCID-5-I/P W/ PSY SCREEN). This comprehensive semi-structured clinical interview is a reliable and valid means to acquire information regarding psychiatric disorders (Diagnostic and Statistical Manual of Mental Disorders, 2013).

#### Self-report measures included in the original protocol

Beck Depression Inventory (BDI-II). This is a psychometrically sound 21-item self-report measure to assess depressive symptoms (Beck et al., 1961; Wang and Gorenstein, 2013).

Harvard Food Frequency Questionnaire (Harvard FFQ) 2007 Booklet. The Harvard FFQ is a comprehensive 101-item psychometrically sound semi-quantitative food frequency questionnaire (Willett et al., 1987).

Insomnia Severity Index (ISI) is a reliable and valid 7-item instrument assessing the nature and severity of insomnia symptoms (Morin, 1993).

Outcome Questionnaire-45 (OQ-45) is a 45-item questionnaire that is designed to measure distress associated with key areas of functioning (e.g., interpersonal functioning, social role) (Lambert et al., 1996).

PTSD Checklist for DSM-5 (PCL-5) is a 20-item self-report measure used to assess PTSD symptom severity, based on DSM-*5* diagnostic criteria (Diagnostic and Statistical Manual of Mental Disorders, 2013).

Rocky Mountain MIRECC Demographics Questionnaire includes standard demographic questions and information regarding military history.

36-Item Short Form Health Survey (SF-36) is a 36-item multi-purpose, short-form health survey (Brazier et al., 1992) that yields an 8-scale profile of functional health and well-being scores.

The following surveys were added 3/12/2018.

#### Self-report measures added to the protocol March 2018

International Physical Activity Questionnaire (IPAQ) Short Form is a reliable and valid 7-item self-report measure of physical activity (Craig et al., 2003).

Morningness/Eveningness Questionnaire (MEQ) is a 19-item self-report instrument designed to assess an individual's chronotype (Horne and Ostberg, 1976).

National Health Interview Survey (NHIS)—Chronic Conditions (National Center for Health Statistics, 1985) is used to query regarding chronic health conditions (Ward and Schiller, 2013).

Neurobehavioral Symptom Inventory (NSI) is a widely-used measure of postconcussive symptoms (Cicerone and Kalmar, 1995).

Patient Health Questionnaire-9 (PHQ-9) is a frequently used and psychometrically sound self-report measure of depression (Kroenke et al., 2002; Pinto-Meza et al., 2005).

Seasonality Pattern Assessment Questionnaire (SPAQ) is a research and screening tool that is widely used in studies of seasonality of mood and behavior, and of Seasonal Affective Disorder (Rosenthal et al., 1987).

#### Self-report measure regarding the built environment added to the protocol March 2018

Housing, Occupancy, Materials, and Environment (HOME) Survey is comprised of questions associated with aspects of the built environment.

### Veteran cohort characteristics

Most of this cohort was male (85.1%). From a demographic standpoint, the sample is relatively diverse. For example, it includes a wide range of ages (24–77 years; average age ± SD, 47.0 ± 13.8 years). Eight percent of the sample reported a sexual orientation other than heterosexual, and a majority pursued education beyond high school (~87%). Perhaps unique to this population, 8.5% of the sample reported current homelessness and 48% reported ever being homeless. While most of the cohort is Caucasian (63.8%), there is greater representation of African Americans (21.3%) and those of Hispanic ethnicity (13.3%) than has been observed in most human microbiome studies. Further details regarding the demographic characteristics are highlighted in Table 2.

**Table 2:** Demographic characteristics.

**Variable**	**N (%) *or* Mean ±SD (range)**
Total	188 (100%)
Age	47.0 ± 13.8 (24–77)
**AGE CATEGORIES**	
20–29	22 (11.7%)
30–39	50 (26.6%)
40–49	25 (13.3%)
50–59	50 (26.6%)
60–69	36 (19.1%)
70+	5 (2.7%)
**GENDER**	
Male	160 (85.1%)
Female	28 (14.9%)
**RACE**	
Caucasian	120 (63.8%)
African American	40 (21.3%)
Asian	3 (1.6%)
Native American	5 (2.7%)
Multiracial	6 (3.2%)
Other	14 (7.4%)
**ETHNICITY**	
Hispanic	25 (13.3%)
Non-Hispanic	163 (86.7%)
**MARITAL STATUS**	
Married	62 (33.0%)
Single	52 (27.7%)
Cohabitating	7 (3.7%)
Separated/Divorced	58 (30.8%)
Widowed	9 (4.8%)
**SEXUAL ORIENTATION**	
Heterosexual	173 (92.0%)
Gay/Lesbian/Queer	9 (4.8%)
Bisexual	4 (2.1%)
Other	2 (1.1%)
**EDUCATION LEVEL**	
No High School Degree	1 (0.6%)
High School Degree	23 (12.2%)
Some College	72 (38.3%)
Associate Degree	26 (13.8%)
Bachelor Degree	50 (26.6%)
Master's Degree	13 (6.9%)
Doctoral Degree	3 (1.6%)
**EMPLOYMENT STATUS**	
Employed Full-Time	48 (25.5%)
Employed Part-Time	17 (9.0%)
Unemployed Seeking Job	40 (21.3%)
Unemployed Not Seeking Job	34 (18.1%)
Retired	49 (26.1%)
**STUDENT STATUS**	
Not in school	153 (81.4%)
Full-Time	27 (14.3%)
Part-Time	8 (4.3%)
**CURRENTLY HOMELESS**	
Yes	16 (8.5%)
No	172 (91.5%)
Number of Times Ever Homeless	1.3 ± 2.6 (0–20)

The military service history of this Veteran sample is also diverse, with all service branches represented, a wide-range of time spent on Active Duty service (3–360 months), and service across multiple eras (Table 3). Of note, 83% (*n* = 156) of the sample met criteria for at least one mental health condition (current and/or lifetime). For a summary of mental health characteristics (as well as data regarding general population prevalence rates for PTSD and mood disorders) see Table 4.

**Table 3:** Military service characteristics.

**Variable**	**N (%) or Mean ±SD (range)**
**SERVICE BRANCH**	
Army	91 (48.4%)
Air Force	36 (19.1%)
Navy	28 (14.9%)
Marines	25 (13.3%)
Coast Guard	1 (0.6%)
Multiple	7 (3.7%)
**SERVICE TYPE**	
Active Duty (AD) only	134 (71.3%)
Reserve Duty (RD) only	4 (2.1%)
AD & RD	50 (26.6%)
Months on Active Duty (*n* = 184)	81.3 ± 67.1 (3–360)
Months on Reserve Duty^a^ (*n* = 53)	63.1 ± 70.0 (2–372)
**SERVICE ERA**	
Post-Korean War	2 (1.1%)
Vietnam War	17 (9.0%)
Post-Vietnam War	44 (23.4%)
Desert Storm	18 (9.6%)
OEF/OIF/OND	63 (33.5%)
Multiple	44 (23.4%)
Year Separated from Service^a^ (*n* = 179)	1997 ± 16 (1959–2017)
**DEPLOYED**	
Yes	132 (70.2%)
No	56 (29.8%)
Number Times Deployed^b^ (*n* = 132)	2.8 ± 2.4 (1–15)
**COMBAT**	
Yes	89 (47.3%)
No	99 (52.7%)
Number Times in Combat^b^ (*n* = 89)	2.0 ± 1.3 (1–8)

a *Some observations had missing data on variables of interest. Specifically, one observation for a Reserve Duty Veteran was missing data on months of service, and 9 observations were missing data for the year separated from service*.

b *Continuous variables on the number of times deployed and the number of times in combat were only reported among those observations with any deployment or combat exposure*.

*AD, Active Duty; OEF, Operation Enduring Freedom; OIF, Operation Iraqi Freedom; OND, Operation New Dawn; RD, Reserve Duty*.

**Table 4:** Prevalence of psychiatric diagnoses.

**SCID-5 disorder category**	**Sample Prevalence** ***n*** **(%)**	
	**Lifetime**	**Current**
PTSD^*^	78 (42.2%)	47 (25.4%)
Other anxiety disorders	35 (18.9%)	26 (14.1%)
Mood disorders^**^	99 (53.5%)	42 (22.7%)
Substance use disorders (Alcohol & Drug)^***^	112 (60.5%)	40 (21.6%)
Schizophrenia psychotic disorders^****^	3 (1.6%)	2 (1.1%)
Eating disorders	5 (2.7%)	2 (1.1%)
Sleep disorders	–	22 (11.9%)
Other SCID-5 disorders^a^^,^^b^	7 (3.8%)	25 (15.5%)

a *Other Lifetime Disorders include: Other Specified Trauma- and Stressor-Related Disorder, Hoarding Disorder, Body Dysmorphic Disorder, and Other DSM-5 Disorder*.

b *Other Current Disorders include: Adult Attention-deficit/Hyperactivity Disorder, Intermittent Explosive Disorder, Other Specified Trauma- and Stressor-Related Disorder, Acute Stress Disorder, Hoarding Disorder, Gambling Disorder, Body Dysmorphic Disorder, Somatic Symptom Disorder, and Other DSM-5 Disorder*.

* *National PTSD prevalence estimates lifetime and past 6 months, 9.4%, 4.2% (Kilpatrick et al., 2013)*.

** US adults past year prevalence, major depressive episode and bipolar disorder, 6.7% and 2.8%, respectively (Center for Behavioral Health Statistics and Quality, 2017)

*** *US national prevalence rates of Drug Use Disorders, lifetime and past 12 months, 9.9% and 3.9%, respectively (sedative/tranquilizer, cannabis, amphetamine, cocaine, nonheroin opioid, heroin, hallucinogen, club drug [e.g., ecstasy, ketamine], and solvent/inhalant use disorders) (Grant et al., 2016). Past 12-month heavy alcohol users, 6.2%, age 12 and older (Center for Behavioral Health Statistics and Quality, 2015)*.

**** *Worldwide 12-month and lifetime prevalence of schizophrenia (median estimate), 0.33% and 0.48%, respectively (Simeone et al., 2015)*.

## Current advances and future directions

The study described above is the first large-scale cross-diagnostic, longitudinal effort focused on the microbiome and US military Veteran health. Veterans enrolled in this study were psychosocially diverse, and reported a notable history of military deployments and service to the nation. Service in conflicts dating back to Vietnam were reported, with the mean number of deployments being almost three. Nearly 50% of those enrolled endorsed a history of combat experience. On the SCID-5 (First et al., 2015), the gold standard for lifetime psychiatric diagnoses, 42.2% of Veterans reported PTSD, 53.5% a mood disorder, and over 60.5% a substance use disorder.

Data being collected also allows for exploration of an understudied area the relationship between the social determinants of health (SDH) and the microbiome. According to the World Health Organization (World Health Organization, 2018) social determinants are conditions in which people are born, grow, live, work, and age, which are influenced by “economic policies and systems, development agendas, social norms, social policies and political systems” that in turn influence health and disease. Frequently studied components of SDH include: economic stability; education; social and community context; health and healthcare; and, the neighborhood and built environment. The health of Veterans has been and continues to be influenced by such factors, some of which certainly predate time in the military. As such, intentional recruitment of a high-risk cohort and collection of microbiome and psychosocial data are expected to increase understanding regarding relationships between individuals and environmental factors known to influence both disease and dysbiosis.

While most previous efforts regarding the microbiome and health have focused on the gut, members of the US-VMP team expanded our focus to include skin and oral microbiomes, as well as the microbiome of the built environment. Although this was at least in part driven by the diverse backgrounds of our team members, the primary motivator is our shared vision regarding the impact of the inner (e.g., human microbiome) and outer (e.g., environment) layers of microbial diversity on human physical and psychological health (Ruokolainen et al., 2017). That is, to understand the “bi-directional *environmental-microbiota-health* axis” it is necessary to focus on the built environment and beyond (Ruokolainen et al., 2017, p. 45). Similarly, this holistic point of view has fostered our thinking regarding innovative connections between seemingly disparate, but likely interrelated, biological systems, as well as microbial and social determinants of health.

Debelius et al. (2016) highlight the importance of turning microbiome research into usable data by implementing systematic methods. The members of this team are committed to such efforts. Moreover, it is our hope that data being collected will become a resource for the scientific community at-large. We foresee that this project will greatly increase our understanding of determinants of the human microbiome, including exposures such as the built environment, diet, supplements, and medications, as well as how the microbiome relates to physical and mental health conditions. This knowledge could help to guide microbiome-related interventions, open new avenues for diagnostics, and increase our understanding of the pathophysiology of disease.

We are mindful of potential obstacles, including “budget restrictions and disease focus by agency,” which create barriers to collecting, processing, analyzing, and sharing data (Debelius et al., 2016). Nonetheless, we assert that just such efforts will be required to lay the foundation to understand how complex exposures help to shape the microbiomes of patients seen in clinical settings, and to explore and potentially identify “core” Veteran microbiomes, with the goal of highlighting both similarities and differences between Veteran and civilian cohorts.

The combination of the extensive EMR, with the collection of psychometrically sound measures of physical and mental health, provides this team, as well as the larger scientific community, with an exceptional opportunity to investigate the role of microbial communities in psychological and physical health and disease. This will require collaborative efforts, as well as a shift in focus from the microbiome to integrative health approaches. Such holistic, patient-focused approaches will address the whole person, by focusing on interactions between individuals' genetics, SDH, microbiomes, health-related behaviors (e.g., sleep, exercise, diet), and mental, physical, and cognitive functioning.

## Author contributions

All of the authors contributed to this article. LB, AH, CL, TP, and KS-Y conceptualized the research. KS-Y, CH, CS, and JH provided data curation. CH and MS provided formal analysis, while LB acquired funding, project administration, and resources. KS-Y and DH conducted the investigations. LB, AH, and CL provided the methodology, and CH the visualization. LB, AH, KS-Y, CS, JH, CH, MS, and CL wrote the original draft, while LB, AH, KS-Y, CS, JH, TP, CH, MS, and CL reviewed and edited it.

### Conflict of interest statement

The authors declare that the research was conducted in the absence of any commercial or financial relationships that could be construed as a potential conflict of interest.
