# Supplementary material for: Military-Related Exposures, Social Determinants of Health, and Dysbiosis: The United States-Veteran Microbiome Project (US-VMP)
Source: Front Cell Infect Microbiol. 2018 Nov 19;8:400. doi: 10.3389/fcimb.2018.00400 (PMC6252388; doi:10.3389/fcimb.2018.00400)
Supplement: Supplementary file 1 [file Data_Sheet_1.docx]

**Supplemental Appendix 1. Sampling Protocols**

Skin Sampling Protocol

1. A research assistant would put on nitrile or latex gloves
2. The research assistant would hand the participant a sealed double tipped sampling swab
3. The research assistant would instruct the participant to open the collection tube to expose the swab
4. The participant was instructed to firmly swab their inner elbow for 10 seconds being careful to avoid contacting anything else with the swab
5. The participant was instructed to place the swabs back into the collection tube
6. The participant would hand the collection tube back to the research assistant
7. The research assistant wrote the date and time on the collection tube label
8. The research assistant would then place the sample into a freezer for storage at ­–80°C

Oral Sampling Protocol

1. A research assistant would put on nitrile or latex gloves
2. The research assistant would hand the participant a sealed double tipped sampling swab
3. The research assistant would instruct the participant to open the collection tube to expose the swab
4. The participant was instructed to open their mouth and swab the inside of one cheek firmly for 10 seconds being careful to avoid contacting the teeth, gums, and tongue
5. The participant was instructed to place the swabs back into the collection tube
6. The participant would hand the collection tube back to the research assistant
7. The research assistant wrote the date and time on the collection tube label
8. The research assistant would then place the sample into a freezer for storage at ­–80 °C

Fecal sampling protocol (In-clinic)

1. A research assistant would put on nitrile or latex gloves
2. The research assistant would hand a sampling kit to a participant, which contained the following instructions

***How to collect your sample****:*

- Take the collection tube to the restroom with you to collect your stool sample.
- Once your stool movement is complete. Put on your gloves and wipe with toilet paper.
- Carefully holding the used toilet paper, open the tube with the swab inside with the other hand. Be careful not to touch the stem or cotton swab.
- Use the swab tip to collect a sample from the toilet paper. You only want to collect enough to color half the swabs brown. Do not fill the entire swab with your sample.
- Place the swab back into the tube
- Remove your gloves, flush the toilet and wash your hands after you have completed collecting the sample.

1. Upon the return of the participant, the research assistant would take the collection tube with soiled swab and write the date on the collection tube label
2. The research assistant would then place the sample into a freezer for storage at ­–80°C

Fecal Sampling Protocol (Out-of-clinic)

Below is the text from an instruction sheet that was provided with the fecal sampling clinic for out-of-clinic use.

***Sampling Protocol for Gut Swab Microbial Sampling***

- Please do not open the kits until after you are in the restroom. Handle the tube by the red cap only.
- You will not collect your entire stool sample. You will be using the swabs to get your sample from the “first use” toilet paper (the first wipe with the toilet paper you make after your stool movement is complete)
- Do not collect your sample until you are sure you can drop the sample in the mailbox on the same day.
- Place the cold pack provided in the freezer.

***How to collect your sample:***

1. Take the 2 collection tubes to the restroom with you to collect your stool sample.
2. Once your stool movement is complete. Put on your gloves and wipe with toilet paper.
3. Carefully holding the used toilet paper, open the tube with the swab inside with the other hand. Be careful not to touch the stem or cotton swab.
4. Use the swab tip to collect a sample from the toilet paper. You only want to collect enough to color half the swabs brown. Do not fill the entire swab with your sample.
5. Place the swab back into the tube.
6. Repeat steps 3-5 with the second tube.
7. Remove your gloves, flush the toilet and wash your hands after you have completed collecting the sample.

***After you have collected your sample:***

1. Record the date and time you collected your sample on the outside of the tube.
2. Put the tubes and the cold pack in the self-addressed envelope provided to you.
3. Seal the envelope.
4. Drop the envelope off at the nearest mailbox or post office.

If something out of the ordinary occurs, like you touch the swab to another surface or drop the swab on the floor, please notify a member of the study team.
